# Intraspecific Variation for Leaf Physiological and Root Morphological Adaptation to Drought Stress in Alfalfa (*Medicago sativa* L.)

**DOI:** 10.3389/fpls.2022.795011

**Published:** 2022-05-03

**Authors:** Silvas Prince, Md Rokebul Anower, Christy M. Motes, Timothy D. Hernandez, Fuqi Liao, Laura Putman, Rob Mattson, Anand Seethepalli, Kushendra Shah, Michael Komp, Perdeep Mehta, Larry M. York, Carolyn Young, Maria J. Monteros

**Affiliations:** ^1^Noble Research Institute, LLC, Ardmore, OK, United States; ^2^BASF, Morrisville, NC, United States; ^3^MLM Medical Labs, Oakdale, MN, United States; ^4^Conservation Technology Information Center, Lafayette, IN, United States; ^5^Biosciences Division and Center for Bioenergy Innovation, Oak Ridge National Laboratory, Oak Ridge, TN, United States; ^6^Department of Entomology and Plant Pathology, Oklahoma State University, Stillwater, OK, United States; ^7^Bayer Crop Science, Chesterfield, MO, United States

**Keywords:** alfalfa, drought stress, root responses, normalized difference vegetation index (NDVI), algorithms

## Abstract

Drought stress reduces crop biomass yield and the profitability of rainfed agricultural systems. Evaluation of populations or accessions adapted to diverse geographical and agro-climatic environments sheds light on beneficial plant responses to enhance and optimize yield in resource-limited environments. This study used the morphological and physiological characteristics of leaves and roots from two different alfalfa subspecies during progressive drought stress imposed on controlled and field conditions. Two different soils (Experiments 1 and 2) imposed water stress at different stress intensities and crop stages in the controlled environment. Algorithm-based image analysis of leaves and root systems revealed key morphological and physiological traits associated with biomass yield under stress. The *Medicago sativa* subspecies (ssp.) sativa population, PI478573, had smaller leaves and maintained higher chlorophyll content (CC), leaf water potential, and osmotic potential under water stress. In contrast, *M. sativa* ssp. varia, PI502521, had larger leaves, a robust root system, and more biomass yield. In the field study, an unmanned aerial vehicle survey revealed PI502521 to have a higher normalized difference vegetation index (vegetation cover and plant health characteristics) throughout the cropping season, whereas PI478573 values were low during the hot summer and yielded low biomass in both irrigated and rainfed treatments. RhizoVision Explorer image analysis of excavated roots revealed a smaller diameter and a narrow root angle as target traits to increase alfalfa biomass yield irrespective of water availability. Root architectural traits such as network area, solidity, volume, surface area, and maximum radius exhibited significant variation at the genotype level only under limited water availability. Different drought-adaptive strategies identified across subspecies populations will benefit the plant under varying levels of water limitation and facilitate the development of alfalfa cultivars suitable across a broad range of growing conditions. The alleles from both subspecies will enable the development of drought-tolerant alfalfa with enhanced productivity under limited water availability.

## Introduction

Drought negatively impacts crop productivity and the long-term persistence of perennial crops (Araus et al., 2002). Understanding drought-adaptive mechanisms used by plants in both aboveground tissues and root systems can provide valuable insights to enhance water uptake and use efficiency. Leaf-based physiological and biochemical traits hold the potential to improve stress adaptation and performance under abiotic stresses (Deshmukh et al., 2014; Prince et al., 2015a). Plant root systems serve additional roles, including anchoring the plant to the ground and providing a substrate for interactions with soil microbes (Paez-Garcia et al., 2015).

Alfalfa (*Medicago sativa* L.) is a perennial outcrossing forage legume species (Hanson et al., 1988), a source of high-quality forage with broad geographical adaptability (Cash and Yuegao, 2009) and is suitable for being grown in different soil types with varying nutrient compositions (Russelle and Lamb, 2011). Selection for specific root-branching traits increased alfalfa biomass production in greenhouse and field evaluations (McIntosh and Miller, 1980; Pederson et al., 1984; Saindon et al., 1991; Lamb et al., 2000). Many researchers have described shifts in root growth and development in alfalfa and other crop species under limited water availability (Lamb et al., 1999; Widrig, 2005; Zhu et al., 2005; Mi et al., 2010). Classical alfalfa studies revealed a selection of fibrous (small diameter) roots and higher lateral root numbers increased biomass under drought stress (Perfect et al., 1987). Further modulation of root physiology through regulation of metabolites confers winter hardiness and fall dormancy (Haagenson et al., 2003).

Challenges in root phenotyping include their underground location and the complexity of root development in response to different growing conditions (Prince et al., 2013). Therefore, most studies have focused on the aboveground responses, and only the limited information is available for root traits with the potential to increase drought stress adaptation. Recent advances in root phenotyping strategies suitable for basic, translational, and practical applications (Paez-Garcia et al., 2015) enhanced our understanding of drought-adaptive mechanisms of alfalfa and addressed current knowledge gaps. Previous studies in alfalfa have identified genetic loci for biomass production under drought stress (Ray et al., 2015; Zhang et al., 2015) and evaluated differences in tissue-specific gene expression and metabolite accumulation in response to drought stress (Kang et al., 2011). Plant phenotyping capabilities always back up crop genetic improvement. Recent advancements in unmanned aerial vehicle (UAV) technology paired with sensors and computer modeling have enabled effective tracking of alfalfa development through its life stages and its response to the environment (Pittman et al., 2015; Cazenave et al., 2019).

The *Medicago sativa* species complex includes the tetraploid subspecies (ssp.) sativa, falcate, and varia. Individuals from *Medicago sativa* ssp. varia tend to have a thicker taproot diameter and higher root biomass under drought conditions and are well-adapted to rangeland conditions (Anower et al., 2017). The *Medicago* ssp. varia individuals have the least productivity, higher persistence, and drought tolerance in low-input management grazing systems than the sativa ssp. However, cultivars developed between varia and sativa ssp. yielded only 84–91% of sativa cultivars (Li et al., 2010). To develop drought stress adapted and yield performance alfalfa cultivars, information on intraspecific diversity for leaf physiology, morphology and root traits becomes crucial for crop improvement. Furthermore, different factors, including natural forces that force differentiation (Hufford and Mazer, 2003), genetic diversity, and the intensity of drought stress and varying environmental conditions, can affect adaptation strategies to drought stress in crop plants (Tardieu et al., 2018). Thus, it is imperative to evaluate alfalfa ssp. responses under variable drought to identify stress adaptive traits and develop stress-resilient alfalfa cultivars. This study was designed to evaluate the morphological and physiological characteristics of roots and shoots from two *M. sativa* ssp. sativa and *M. sativa* ssp. varia alfalfa under progressive drought and rainfed conditions imposed on controlled mesocosms and in the open-field, respectively. This combinatorial experiment facilitated the identification of key leaf and root traits (morphological, physiological, and plastic response) and their combinations that influence biomass production under water limitation in alfalfa.

## Materials and Methods

### Plant Materials

The alfalfa germplasm selected for this study (Supplementary Table 1) were *M. sativa* ssp. sativa PI478573 (originating from Lima, Peru, in a mild desert climate), *M. sativa* ssp. varia PI502521 (originating from the former Soviet Union with cold and prolonged winters), along with a synthetic alfalfa variety, Bulldog805, released by the University of Georgia (*M. sativa* subsp. sativa PI594913). The alfalfa variety, Bulldog805, is created by random intermating of desirable population of plants, and the seed collected will be identified as a synthetic variety. The germplasms, PI478573 and PI502521 focused here, are genetically diverse to identify desired traits such as drought tolerance and biomass yield.

### Greenhouse Drought Experiment

The seed of selected alfalfa was scarified using 3M Paper Sheet 210N and surface-sterilized with a 2.5% bleach solution (3 min) followed by three washes in deionized water. The sterilized seed was placed in deionized water overnight at 4°C and transferred to a Petri dish-lined with moistened filter paper (Whatman No. 1) at 24°C dark to induce germination. After 4 days, the germinated seedlings were transplanted to soil media in the PVC columns (76.2 cm length, 15.24 cm diameter) lined with a 3 MIL plastic sleeve (Uline, Pleasant Prairie, WI, United States). The soil media contains 43 g of Osmocote fertilizer (N:P:K composition of 15:9:12)/gallon. Two PVC mesocosms (experimental ecosystems closest to the real world), Exp. 1 and Exp. 2, were conducted in a single layout in the greenhouse with two treatments, well-watered (WW) and water-stressed (WS). In Experiment 1 (Exp. 1), the top 15 cm of the PVC column consisted of Metro-Mix 360 (Sun Gro, Agawam, MA, United States), and the bottom (61 cm) contained All-Purpose sand (Quikrete, Atlanta, GA, United States). In Experiment 2 (Exp. 2), the soil medium in the PVC column was a sand and perlite mixture [of 2:1 (v:v)]. Each experimental layout (Exp. 1 and Exp. 2) was a randomized complete block design with four replications (four individual plants were considered as four replicates as the alfalfa seeds are heterogeneous, i.e., each seed is genetically diverse) arranged in four blocks with 6 PVC columns/block with WW and WS treatments labeled with green and white pot labels, respectively (Supplementary Figure 1).

Plants were grown at a light intensity of 650 μmol m^–2^ s^−1^ and a relative humidity of 68%. In each experiment, 24 PVC columns were used (3 genotypes × 4 replications × 2 treatments). Further details on the irrigation and drought stress experiment are listed in Supplementary Table 2. In both experiments, each PVC columns were top-watered daily with 50 ml of tap water for 18 days after transplanting (DAT), starting on 2 September 2015. To monitor soil moisture depletion, two EC-5 soil moisture sensors (METER Environment, Pullman, WA, United States) were placed at 15 and 60 cm depths at the rate of six sensors (2 depth of sensors × 3 PVC columns per treatment) to track every 15 min automatically logged into CR1000 data loggers (Campbell Scientific, United States). The above-ground biomass was harvested in both Exp. 1 and Exp. 2 four weeks after drought stress. A recovery period of 21 days in both experiments was established by top watering plants with 150 ml of tap water once a week for 3 weeks followed by a second above-ground biomass harvest and terminal root biomass harvest at the end of the drought recovery period. The biomass samples were oven-dried to get dry weight biomass data. The fresh and dry weights of both shoots and roots after harvesting were determined in grams using a balance.

### Physiological Response of Leaf Tissues Under Drought

After water stress imposition, measurements on the uppermost fully expanded trifoliate for CC with the SPAD 502 Plus Chlorophyll Meter (Spectrum Technologies, Aurora, IL, United States), stomatal conductance (gs), osmotic potential (OP), and leaf water potential (LWP) were measured. The gs of the middle leaflet from the first fully expanded trifoliate was measured with a LI-6400 photosynthesis system (LI-COR, Lincoln, NE, United States) under light intensity set at 500 mmol m^2^s^–1^ and flow rate at 400 μmol s^–1^. The OP was determined using the detached uppermost trifoliate leaf samples immediately placed in a test tube (Pyrex, New York, NY, United States) with deionized water sealed with a rubber stopper and stored overnight at 4°C. A 7–10 μl aliquot of the leaf sap was transferred to a filter paper disk fitting the C-52 Wescor sample chamber, and the OP was measured using a Wescor HR-33T microvoltmeter (Wescor Inc., United States) operating in the “dew point” mode. Multiple readings per leaf were determined until the values stabilized at less than 0.04 MPa. The LWP before and after the water stress was determined using an L-51 PSYPRO thermocouple psychrometer (Wescor Inc., United States) 7 days after water was withheld in the WS treatment and 24 h after the plants were rewatered at the completion of the WS treatment.

### Acquisition and Image Processing of Alfalfa in Greenhouse

In Exp. 1, a total of four fully developed meristematic trifoliate leaves from each replicate were scanned using the Epson Perfection V33 scanner (Epson America Inc., United States) at 600 dpi with a white background and processed using custom MATLAB (2012–2016, The MathWorks Inc., United States) scripts (Paez-Garcia et al., 2019). In Exp. 2, the leaf images were collected using a Canon camera EOS 7D (lens EF-S 50 f/1.2L USM) with a black background and a ruler with metric units for size calibration. Standardization of the image size was based on the pixel counts that are represented in 1 cm spacing using the “image size” function in Photoshop (Adobe, San Jose, CA, United States). Measurements on total blade area, blade width, length, length-to-width ratio, compactness, and entropy (Neto et al., 2006) were made on the three individual leaflets (left, center, and right) of fully matured trifoliate as described for the LeafletAnalyzer program (Liao et al., 2017). Before the measurements, the white canvas replaced the background of the leaf image (Supplementary Figure 2), and MATLAB scripts integrated into the program determined the leaf morphology, shape, and size (Supplementary Figure 3). Next, we visualized the distribution of alfalfa studied in a three-dimensional morphospace confined by three blade width, total blade area, and length-width ratio parameters selected based on leaf morphological classification with the Leaflet Analyzer (Liao et al., 2017).

Roots collected from the PVC mesocosms were washed with a fine flow of running water, blotted dry, and photographed using a Canon EOS 7D camera with EF-S 50 f/1.2L USM lens (Canon Inc., United States). Photographs in TIFF format were analyzed using the WinRHIZO Pro software (v.2009, Reagent Instruments, Canada) to collect data on root diameter. Additional data on root phenotypic traits like root width and root angle at four different segments (each segment makes up 25% of total root length), according to quartiles of total root length based on total root area, and vertical root length were extracted using custom MATLAB scripts (Paez-Garcia et al., 2019).

### Raised Bed Field Experiment

To address the heterogeneity and to evaluate a higher number of plants, three accession/cultivar evaluated in a greenhouse [PI478573, PI502521, and Bulldog805] were planted in raised beds replicated three times with five plants per replication following a spaced planting method (10 inches apart) on 15 September 2016. These accessions were included in another experiment evaluating six alfalfa check cultivars for their biomass yield under water limitation and persistence. All three accession/cultivar were grown in two raised bed representing two treatments, rainfed and irrigated conditions. The raised beds were fabricated using 10-inch c-purlin with approximate 16 feet × 8 feet × 20 (length × breadth × height) inches, with the bottom that has approximately 2 inches of 1/4-inch pea gravel to aid drainage and is filled with ‘‘Okie Dirt,’’ a mixed composted material^1^. The inner walls of the bed were sprayed with 2 inches of spray foam to avoid heating of the soil within the elevated walls.

In both beds, the soil moisture sensors were installed at two different depths, 10 and 25 cm, with six sensors/bed to collect soil moisture on a volume basis. The first biomass harvest was made on 1–2 May 2017, with subsequent four harvests made at an interval of 30 days on 6–7 June, 7–8 July, 3–4 August, and 11–12 September 2017. After the fourth biomass was collected, one side of the raised bed wall was removed to excavate the root system using a shovelomics approach on 11–15 September 2017 and imaged using the camera-based RhizoVision Crown platform (Seethepalli et al., 2020) and analyzed using RhizoVision Explorer (Seethepalli et al., 2021).

### Unmanned Aerial Vehicle Data Collection and Processing

A DJI Inspire 1 UAV equipped with a DJI Zenmuse X5 visual band sensor camera (resolution 0.6–1.1 cm per pixel for RGB images) and a Sentera single sensor multispectral camera (resolution 2.5 cm per pixel for NDVI images) was deployed for normalized difference vegetation index (NDVI) data collection as described in Cazenave et al. (2019). Images from the UAV were collected at an altitude of 29 m flown at solar noon to minimize the impact of shadows during the image acquisition process, and flown only when the wind speed was below 32 km h^–1^. From January 2017 to September 2017, 17 flights were made to monitor the plant’s growth and performance over the cropping season. The individual UAV-generated images were stitched together using AgiSoft Photoscan Professional version 1.2.2 software (Agisoft LLC, St. Petersburg, Russia) to create orthomosaic images corresponding to the entire raised bed site. The stitching process involves aligning photos and building a dense point cloud, mesh surface, and orthomosaic with high accuracy and high image quality parameters. Finally, the orthomosaic images were geo-referenced using ArcGIS ArcMap software version 10.3.1 (ESRI) by integrating the GPS coordinates from five ground control points positioned both at the center and in each corner of the raised bed site. Initially, a fishnet grid, including the identification of plant number and replicate number, was created using the ArcGIS software to match the data from each row and column that was extracted from the collected image. Using the Zonal Statistic tool in ArcGIS, the fishnet grid was then applied to the masked image to extract the number of pixels as well as the total area covered by these pixels for each plot row directly into a spreadsheet. The total pixel area corresponds to the plant’s ground coverage for further analysis.

### Data Analysis

In Exp. 1 and Exp. 2, the phenotypic data measured on leaf physiological traits (Chlorophyll content, stomatal content, osmotic potential, and leaf water potential), shoot and root fresh, and dry biomass were analyzed using the GLM approach with “blocks” as a fixed variable. Step-wise regression and correlation analyses between traits measured were also performed using SAS version 9.1. The image-based analysis of alfalfa three leaflet blade (area, length, width, entropy, and compactness) and root (diameter, volume, width, angle, and vertical root length) traits were performed using methods described earlier (Liao et al., 2017; Paez-Garcia et al., 2019). Analysis of variance (ANOVA) of shoot (NDVI) and root traits (median, maximum number of roots, total root length, max width, network area, solidity, perimeter, average radius, volume, surface area, maximum radius, steep angle frequency, and holes) from raised-bed experiment was analyzed using ProcGLM analysis in SAS version 9.1, and their relationships between traits were performed using SAS JMP version 15 program. In all the experiments, the least significant difference (LSD) at α = 0.05 was used to determine significant differences among genotypes, treatments, and the interaction. Statistical significance was based on a *p*-value of 0.05.

## Results

### Effect of Water Availability on Alfalfa Leaf Physiology and Shoot Biomass

The two alfalfa plant introductions (PIs) and the Bulldog805 cultivar were grown in two independent experiments with different soil media to study the whole plant’s drought response in alfalfa (Supplementary Table 2). The nature of the soil media used (Metro-mix 360 in Exp. 1 and sand-perlite mix Exp. 2) influenced the water infiltration rate and altered the water availability at different soil depths (Supplementary Figure 4).

**FIGURE 1:**
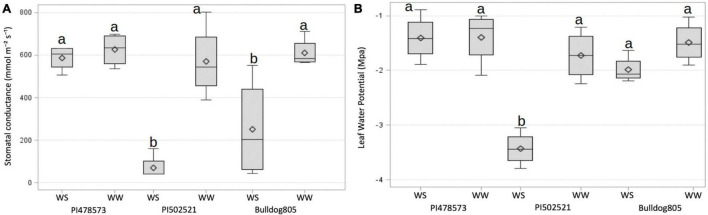
Stomatal conductance **(A)** and leaf water potential **(B)** of plants grown in well-watered (WW) and water-stressed (WS) conditions of Exp. 1. The significance was based on LSD (α=0.05) value for the treatment level. The horizontal line and symbol in the box interior represents the group median and mean phenotypic values with the low and higher Quartile represent as whiskers below and above bar graphs.

After the imposition of water stress, the WS-PVC columns of Exp. 1 experienced a gradual depletion of soil moisture at 15 cm sensor depth to reach volumetric soil moisture of ∼3% at the end of the experiment, whereas the depletion of soil moisture at 60 cm decreased at a faster rate (Supplementary Figure 4A). In Exp. 2, the WS treatment PVC columns experienced a similar pattern of soil moisture depletion at both 15 and 60 cm depth compared to its counterpart, WW treatment PVC columns (Supplementary Figure 4B). After water stress in Exp. 1, the PI478573 regulated photosynthetic activity under stress by maintaining 89.3 and 35% higher gs in comparison to PI502521 and Bulldog805, respectively (Table 1 and Figure 1A). Increase in OP value to 1,081.30 mmol/kg (after water stress) was highly pronounced in PI502521 (Table 1) in contrast to PI478573 and Bulldog805 (Figure 1B and Table 1). The water stress had a significant effect on leaf CC in Exp. 2 by increasing the phenotypic value of PI478573.

**TABLE 1:** Physiological and biomass traits of the aboveground shots and root tissues from three alfalfas evaluated in Exp. 1.

Trait (units)	PI478573	PI502521	Bulldog805	LSD (α = 0.05)	Genotype (G)	Treatment (T)	G x T
Chlorophyll content after water stress (%)	60.99a	54.18a	54.24a	10.80	NS	NS	NS
Stomatal conductance after drought (mmol m^–2^ s^–1^)	606.35a	320.28b	430.93b	138.15	**	***	*
Osmotic potential after water stress (mmol/kg)	582.50a	1081.30a	692.00a	434.16	NS	*	*
Leaf water potential after water stress (Mpa)	–1.40a	–2.58b	–1.74a	0.41	***	***	**
Shoot fresh weight after water stress (g)	17.90a	22.53a	18.70a	7.91	NS	*	NS
Shoot dry weight after water stress (g)	4.83a	5.84a	5.46a	2.43	NS	NS	NS

Significance at genotype, treatment, and genotype × treatment interaction levels were calculated using t-test at p-value (p < 0.0001). Trait means with the same letter indicates no difference between them based on LSD (α = 0.05) value. *, **, *** denotes significance at P < 0.05, 0.001, 0.0001 respectively and NS refers to no significant difference.

**FIGURE 2:**
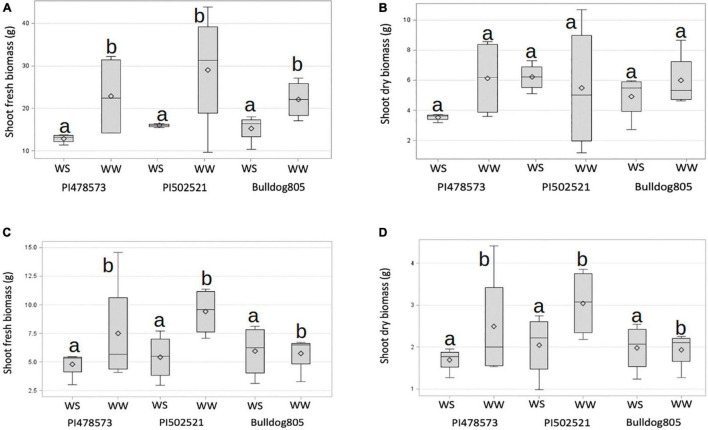
Shoot biomass of alfalfa populations grown under well-watered (WW) and water-stressed (WS) conditions. Variation observed across treatments in Exp. 1 for shoot fresh **(A)** and dry weight **(B)**. Variation observed across treatments in Exp. 2 for shoot fresh weight **(C)** and dry weight **(D)**. The horizontal line and symbol in the box interior represents the group median and mean phenotypic values with the low and higher Quartile represent as whiskers below and above bar graphs.

The fresh and dry shoot biomasses of the three-alfalfa populations were evaluated in Exp. 1 (Figures 2A,B). Limited water availability in the topsoil (15 cm) in Exp. 1 had little effect on average plant biomass produced, compared to Exp. 2 (Figure 2). The maximum shoot fresh biomass of PI502521 under the WW conditions of Exp. 1 was almost 40 g, whereas the maximum yield of a single plant in Exp. 2 was below 12 g (Figure 2C). PI478573 and PI502521 had overall higher fresh biomass yield than Bulldog805 under WW conditions in Exp. 1 (Figure 2A) and Exp. 2 (Figure 2C). Irrespective of variability in water availability in 15 and 60 cm depths within PVC and duration of drought stress imposed, PI502521 had a higher biomass yield under WS treatments in Exp. 1 and Exp. 2 (Figures 2B,D), than other genotypes evaluated.

### Image-Based Assessment of Drought Response in Leaves

**FIGURE 3:**
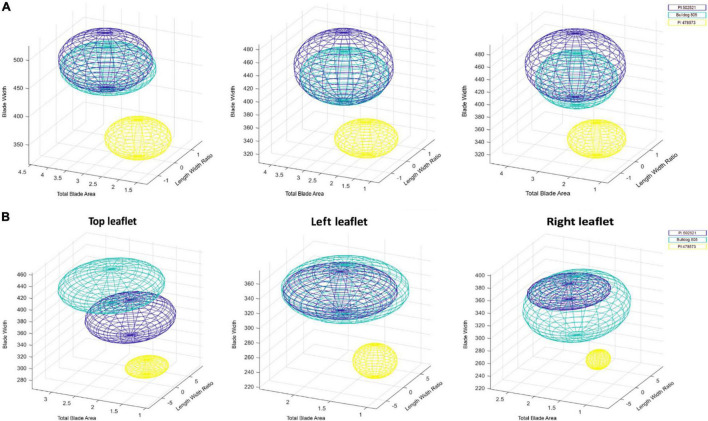
Distribution in the three-dimensional morphospace analysis of leaf groups in alfalfa. Distribution of top, left and right leaf features of alfalfa, PI502521 (dark blue), Bulldog805 (aqua), and PI478573 (yellow) in three-dimensional morphospace defined by blade width, total blade area, and length to width ratio evaluated in Exp. 1 **(A)** and Exp. 2 **(B)** under water stress.

Understanding the response of leaf morphological traits to water stress is critical to improve the photosynthetic efficiency and increase biomass under drought in alfalfa. The first fully matured trifoliate leaf (right, middle, and left) drought responses in morphological traits were quantified in Exp. 1 and Exp. 2 with statistically significant traits listed in Supplementary Table 3. Under WS in Exp. 1, significant spatial leaflet variation (Supplementary Figure 5 and Supplementary Table 3) was observed for leaf morphological traits: total blade area (middle and left), blade area-to-length ratio (left), blade width, and length-to-width ratio (all). The accession, PI478573, had the lowest phenotypic values for total blade area, blade area-to-length, and blade width, followed by Bulldog805 and PI502521. Visualization of leaf features in a three dimensional morphospace exhibited PI478573 to have a distinct space (shown in yellow) confined by blade width, total blade area, and length-to-width ratio. Similarly, the PI502521 (shown in dark blue) and Bulldog805 (shown in aqua) also occupied a distinct space in 3D morphospace (Figure 3). Interestingly, the space occupied by three alfalfa genotypes appeared to be different and consistent with the differences in blade width, total blade area, and length-to-width ratio parameters observed among the genotypes evaluated (Supplementary Table 3).

Differences in leaf morphological traits (blade width, total blade area, and length-to-width ratio) at WS (Supplementary Table 3) affected differences in shoot biomass significantly in Exp. 1 (Table 1). A significant positive association was identified between fresh shoot biomass after water stress and the width of leaflets in a trifoliate, whereas a negative association was identified with leaf length to width ratio in Exp. 1 (Supplementary Figure 6A).

With the higher stress intensity observed in Exp. 2, significant variation was observed for additional leaflet morphological traits identified in Exp. 1 like entropy and compactness (Supplementary Table 3). Irrespective of the difference in the experimental condition, the visualization revealed PI478573 (Figure 3B and Supplementary Table 3) to have a lower phenotypic value for blade width and length-to-width ratio and leaf compactness (middle, left, and right).

### Role of Root Traits in Alfalfa Shoot Biomass Under Drought

Limited water availability at topsoil at 15 cm in Exp.1 and depletion at both depths (15 and 60 cm) at later stages of crop growth in Exp. 2 had a significant impact on shoot fresh biomass yield (Tables 1, 2). The impact of water limitation was highly pronounced in Exp. 2 more than Exp. 1, which is evident from the higher reduction of shoot fresh biomass values ranging from 31 to 34% in contrast to Exp. 1. Irrespective of the difference in stress intensity experienced in Exp. 1 and Exp. 2, PI502521 recorded higher significant values for biomass yield after water stress and recovery from stress (Tables 1, 2). The PI502521 recovered quicker with higher shoot biomass through deeper and thicker root system through allocation of energy and protein reserves in the roots, which is evident from higher root fresh weight and root-shoot ratio (Table 1).

**TABLE 2:** Physiological and biomass traits of the above ground (shoots) and root tissues from three alfalfas evaluated in Exp. 2.

Trait (units)	PI 478573	PI 502521	Bulldog 805	LSD (α = 0.05)	Genotype (G)	Treatment (T)	G x T
Chlorophyll content after water stress (%)	58.43a	49.22b	54.75ab	5.76	*	**	*
Stomatal conductance after water stress (mmol m^–2^ s^–1^)	273.6a	489.4a	342.4a	263.52	NS	NS	NS
Osmotic potential after water stress (mmol/kg)	704.85a	664.17a	669.83a	87.12	NS	NS	NS
Leaf water potential after water stress (Mpa)	–2.08a	–2.18a	–1.91a	2.00	NS	NS	NS
Shoot fresh weight after water stress (g)	6.15a	7.56a	5.85a	3.81	NS	*	NS
Shoot dry weight after water stress (g)	2.09a	2.59a	1.96a	1.07	NS	*	NS

Significance at genotype, treatment, and genotype × treatment interaction levels were calculated using t-test at p-value (p < 0:0001). Trait means with the same letter indicates no difference between them based on LSD (α = 0.05) value. *, ** denotes significance at P < 0.05, 0.001 respectively and NS refers to no significant difference.

**FIGURE 4:**
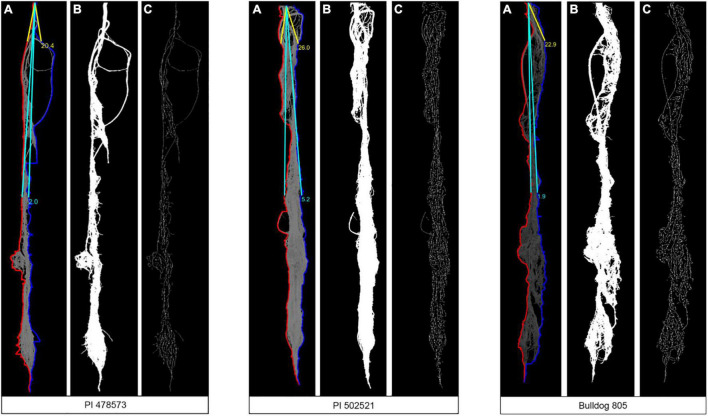
Sample images used for the MATLAB script to evaluate root angle **(A)**, root area **(B)**, and total root length **(C)** of three-alfalfa populations under water-stressed (WS) condition after rewatering plants. Sample images are from plants grown in Exp. 1.

Water limitation experienced in the early crop stage in Exp. 1 significantly influenced the root phenotypic values for diameter (RD), volume (RV), and vertical root length (VRL). Whereas the lateral root angle (LRA) exhibited a significant difference among alfalfa genotypes evaluated in Exp. 2 (Table 3). The PI502521 and PI478573 had the highest and lowest values for RD, RV, and LRA in Exp. 1 and 2 and VRL in Exp. 2, respectively (Table 3 and Figure 4). Among the root traits measured, only the root width (RW) exhibited significance at treatment level (Table 3) with on par phenotypic values between PI502521 and Bulldog805. The cultivar Bulldog805 recorded the highest VRL in Exp. 1 and might have responded to access moisture available at 60 cm soil profile by accumulating deeper root close to the bottom of the PVC column. The shoot biomass after drought exhibited a positive association with RV in Exp. 1 and RW and VRL in Exp. 2 (Supplementary Figures 6A,B).

**TABLE 3:** Phenotypic characterization of root traits in the alfalfa PI478573, PI502521, and Bulldog805 evaluated under water-stressed conditions in Exp. 1 and Exp. 2 evaluated using PVC mesocosms.

Trait, abbreviation (units)	Experiment	PI 478573	PI 502521	Bulldog 805	LSD (α = 0.05)	Genotype (G)	Treatment (T)
Root Diameter, RD (mm)	1	6.06b	8.70a	7.33ab	1.43	*	NS
	2	8.40a	8.98a	7.96a	1.98	NS	NS
Root Volume, RV (cm3)	1	146.99b	293.49a	247.51a	89.59	*	NS
	2	180.23a	282.36a	204.44a	108.91	NS	NS
Root Width Segment I, W	1	117.34a	128.24a	127.28a	46.74	NS	NS
	2	93.41a	130.18a	130.43a	45.88	NS	*
Angle between left soil plane and right most lateral root- Segment III, LRA	1	90.58a	92.22a	92.20a	2.81	NS	NS
	2	92.04ab	92.90a	90.93b	1.49	*	NS
Vertical root length, VRL	1	3,105.4b	3,200.9ab	3,346.8a	185.73	*	NS
	2	2,695.9a	3,050.5a	3,003.1a	285.59	NS	NS

*Significance at genotype, treatment, and genotype × treatment interaction levels were calculated using t-test at p-value (p < 0.0001).*

Trait means with the same letter indicates no difference between them based on LSD (α = 0.05) value. * denotes significance at P < 0.05 and NS refers to no significant difference.

### Field Response of Alfalfa Shoot and Roots Under Water Limitation

**FIGURE 5:**
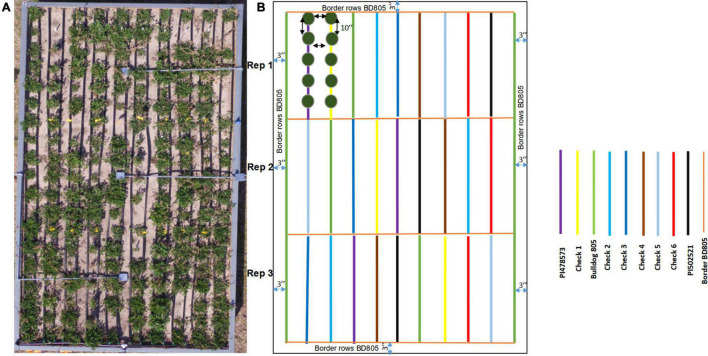
The raised bed experimental plot to study alfalfa response to rainfed treatment. **(A)** Actual plot (Bed 1) to show the details of spaced planting of three alfalfas evaluated in the study along with six check cultivars. **(B)** Field design to explain the spacing, randomization, and replication details. The genotypes and the checks evaluated are color-coded on the legend.

**FIGURE 6:**
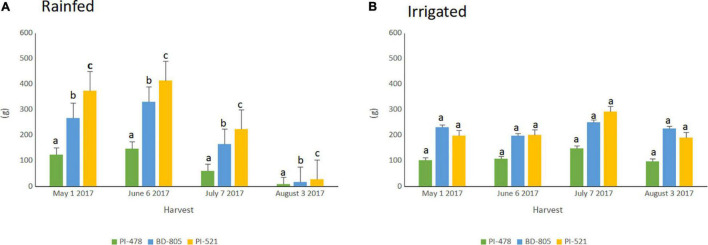
Fresh biomass weight (g) recorded over four harvests within growing season of 2017 among three alfalfas spanning from May to August in irrigated **(A)** and rainfed **(B)** treatments. PI-478; PI478573; BD-805; Bulldog-805 and PI-521;PI502521. Means with different letter indicates biomass yield difference between alfalfa evaluated based on LSD (Alpha-0.05 value).

The selected three alfalfa genotypes were grown in the raised bed (Figure 5) and monitored using NDVI captured throughout the cropping season in 2016 and 2017 (Supplementary Figure 7). Significant variation was observed for NDVI measured across the 2017 cropping season in alfalfa recovered after the 2016 winter season (Table 4). Faster spring recovery was observed in PI502521 and Bulldog805 than PI478573, which corroborates with NDVI values recorded in January and February 2017 (Table 4 and Supplementary Figures 7A,B). The population PI502521 NDVI value was on par with the Bulldog805 throughout the cropping season, whereas the PI478573 NDVI value was low during hot summer months within the cropping season (Supplementary Figure 7B). Repeated measures on dry shoot biomass from four harvests revealed genotypic differences exist between three populations at both treatments. PI502521 population produced higher biomass in the rainfed condition, followed by Bulldog805 and PI478573 (Figure 6A). In the irrigated condition, the PI502521 population also produced a similar biomass yield to the cultivar, Bulldog805. The response on shoot biomass after drought corroborates with the response observed in the greenhouse study was very promising to be used as a proxy to evaluate drought response in alfalfa.

**TABLE 4:** Phenotypic variability of NDVI values measured in 2017 growing season.

NDVI#	Date	Geno	Treat	Geno*Treat
NDVI1	20170127	**<0.0001**	0.1406	0.5342
NDVI2	20170209	**0.0004**	0.8659	0.7602
NDVI3	20170223	**0.0364**	**0.0401**	0.2350
NDVI4	20170309	0.0898	**0.0266**	0.6995
NDVI5	20170328	0.3536	**0.0085**	**0.0363**
NDVI6	20170406	0.4163	**0.0097**	0.8290
NDVI7	20170411	0.7127	0.2400	0.7759
NDVI8	20170428	0.8492	**0.0248**	0.1251
NDVI9	20170505	**0.0158**	**0.0024**	0.3664
NDVI10	20170518	0.0672	**<0.0001**	0.2426
NDVI11	20170615	**0.0241**	0.2674	0.6052
NDVI12	20170705	0.4204	**0.0008**	0.4793
NDVI13	20170713	0.1184	0.7520	0.1917
NDVI14	20170727	0.1540	**<0.0001**	0.1242
NDVI15	20170818	0.2320	**0.0597**	0.5263
NDVI16	20170831	0.3953	0.6428	0.5710
NDVI17	20170907	0.8701	0.4314	0.3695

*Significance at genotype, treatment, and genotype × treatment interaction levels were calculated using t-test at p-value (p < 0.0001).*

*The significant p-values are shown in bold fonts.*

**FIGURE 7:**
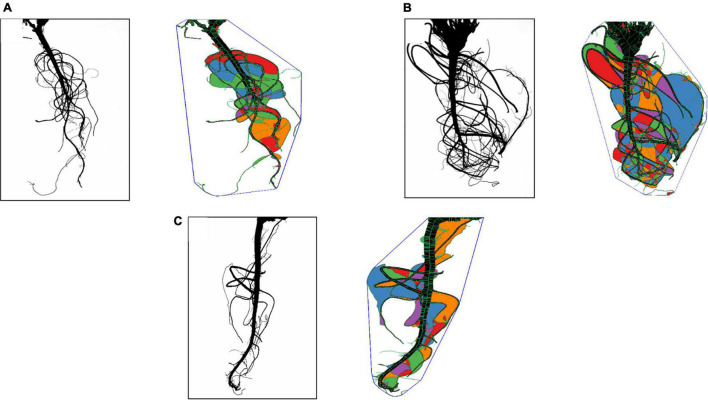
Root traits of alfalfa populations in rain-fed conditions were analyzed using the Rhizovision program. Representative of camera captured and Rhizovision analyzed images of PI478573 **(A)**, PI502521 **(B)**, and Bulldg805 **(C)** are shown.

**FIGURE 8:**
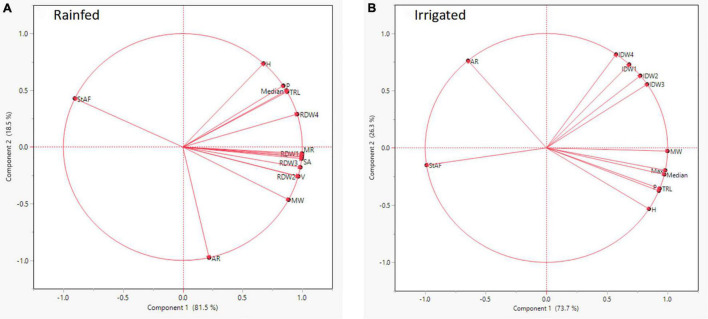
Correlation of statistically significant root architectural traits identified in rainfed **(A)** and irrigated **(B)** conditions with dry biomass yield in alfalfa populations evaluated.

Root segmented image analysis revealed PI502521 had branched root system with higher phenotypic values for most of the root features measured than other alfalfa evaluated (Table 5). The topological image (Figure 7) revealed the PI502521 (Figure 7B) to have higher root volume (represented by blue line), maximum width, and number of roots (green line) key traits to forage water efficiently under limited availability. Extensive measurement on 24 root architecture revealed traits like median, the maximum number of roots, total root length, max-width, network area, surface area, and maximum radius showed a positive association with biomass yield irrespective of treatments across multiple harvests (Supplementary Figures 8A,B). High-yielding alfalfa genotype, PI502521 across treatments, had higher phenotypic values for all root phenotypic traits (Table 5) that were associated with biomass yield. Root traits related to root thickness and angle (like diameter [D], average radius [AR], medium [MAF] and steep [StAF] angle frequencies, fine [FRF], medium [MRF] and coarse [CRF] radius frequencies, and FI-fineness index) were negatively associated with NDVI values (Supplementary Figure 8) and biomass yield (Figure 8) in rainfed condition Irrespective of the treatments, root traits such as diameter, angle frequencies (MAF and StAF), and radius frequencies (MRF and CRF) were negatively associated with biomass yield in alfalfa.

**TABLE 5:** Variability in root architectural traits of alfalfa studied in rainfed (with only rainfall as irrigation source) and irrigated conditions.

Trait (Abbreviation)	Rainfed				Irrigated			
	PI478573	PI502521	Bulldog 805	LSD	PI478573	PI502521	Bulldog 805	LSD
Median (M)	5.30b	8.40a	5.93b	1.78*	4.71b	8.14a	5.00b	1.50*
Max no of roots (Max)	17.15b	22.00a	18.53b	4.35ns	16.50b	21.57a	16.86b	4.50*
Total root length (TRL)	4,209.09a	2,959.30b	2,604.94b	1,012.4*	2585.49b	3642.13a	2506.29c	941.88*
Max width (MW)	158.42b	166.05a	118.44c	30.51*	150.03b	193.24a	159.90c	29.29*
Network area (NA)	6,434.11a	5,845.02b	3,884.07c	1,735.9*	4169.64a	4902.55a	5022.45a	1536.5ns
Solidity (S)	0.19b	0.23a	0.22a	0.04*	0.18a	0.17a	0.19a	0.04ns
Perimeter (P)	3827.37b	5972.04a	4160.09b	1367.3*	3759.50b	5352.69a	3585.35b	1326.2*
Average radius (AR)	1.0015b	1.0199b	1.3504a	0.166*	1.1111b	0.8865b	1.3629a	0.233*
Volume (V)	19704c	41540a	39952b	16923*	23222a	23075a	32488a	14063ns
Surface area (SA)	16398c	27858a	25054b	8293.5*	17642a	20408a	21418a	7480.7ns
Maximum radius (MR)	6.9445c	10.0396a	9.2190b	2.057*	7.5959a	7.7111a	8.7113a	1.6825ns
Steep angle frequency (StAF)	0.5097a	0.4305b	0.4237b	0.0676*	0.5035a	0.4006c	0.4583b	0.0815*
Holes (H)	201.00b	357.87a	180.00c	102.6*	178.93b	303.79a	134.93c	99.75*

*Trait means with the same letter indicates no difference between them based on LSD (α = 0.05) value.*

## Discussion

Responses to drought stress are complex and often involve a range of morphological and physiological adaptations to enhance water uptake and use with optimized root system or reduction in the rate of plant growth as a strategy to conserve water (Annicchiarico et al., 2015; Mutava et al., 2015). Breeding drought tolerance depends on modulating its three major components: dehydration avoidance, tolerance, and drought recovery, which involves a complex interaction among physiological, morphological, and biochemical traits (Luo, 2010). The outbreeding mating system and its ploidy level made conventional breeding challenging tasks in improving various agronomic traits in alfalfa (Zhou et al., 2011) was reviewed recently by Stacy et al. (2018). Crop wild relatives are viewed as novel genetic resources and identified key traits to improve stress resilience in other major legume species (Manavalan et al., 2015; Prince et al., 2015b,^2020^; Beena et al., 2021). In this study, different alfalfa genotypes adapted to a mild desert climate (PI478573) and a cold region with a prolonged winter (PI502521) along with cultivar Bulldog805 were evaluated to identify beneficial leaf and root traits in alfalfa to increase productivity under water limitation.

Leaves, being the primary site of photosynthesis and transpiration, contribute to drought tolerance mechanisms in crop plants. He et al. (2019) revealed leaf area, length, and width as key traits to improve yield and quality and identified to be highly influenced by genotype, environment, and their interaction in alfalfa. Modulation in leaf area is influenced by different factors as genetics, water availability, and abiotic stress factors (Nicotra et al., 2011). Development in studying the leaf responses to drought stress with the use of high-throughput image analysis enabled the integration of stress adaptive strategies to address climate change (Cope et al., 2012). PI478573 has a smaller leaf size compared to the other two populations evaluated. Plants with smaller leaves are better adapted to dry conditions and are often favored under low rainfall conditions (McDonald et al., 2003) to lower evapotranspiration rates and tissue dehydration (Liu and Stützel, 2002). Reduction in the leaf area increased yields in soybean (Prince et al., 2017) and cereal crops (Kadam et al., 2015) under stress. In contrast, the PI502521 had bigger leaves with higher phenotypic values for the area, width, length to width, and area to length ratios and maintained key leaf physiological trait, stomatal conductance, which could potentially increase biomass yield. Differences in leaf morphological traits at WS affected differences in shoot biomass significantly in Exp. 1. A similar contrary response in leaf size was reported in Nordic-type alfalfa that had a higher leaf area than a Morocco ecotype under drought stress (Erice et al., 2010). He et al. (2019) reported leaf area traits as targets in alfalfa breeding to improve yield through enhancing leaf morphology leaf length, area, and width with increasing photosynthetic efficiency.

Despite the reduction in leaf morphological traits, PI478573 maintained higher chlorophyll content, stomatal conductance, leaf water potential, and osmotic potential under drought stress. Similar, enhanced chlorophyll content coupled with a reduction of shoot biomass was reported in a naturalized alfalfa population adapted to a water-limited environment (Anower et al., 2015). A similar response in chlorophyll content was reported in rice among drought-tolerant plants in contrast to their susceptible counterpart (Prince et al., 2015a). The genotype PI478573 was able to maintain higher concentrations of osmolytes in leaves to maintain a positive water gradient in plant tissues. The accumulation of osmoprotectants under stress maintains cell turgor pressure in the leaves and stems, consistent with higher osmotic potential (He et al., 2012). PI502521 has bigger leaves that might increase biomass yield; it also has a higher surface area that could increase the water loss through evapotranspiration. However, PI502521 was found to maintain lower stomatal conductance after drought stress in both greenhouse experiments. Similar genetic ability to maintain stable levels of stomatal conductance was associated with drought-tolerant soybean (Mutava et al., 2015) and rice (Prince et al., 2015a).

The root-shoot communication determines the whole plant’s drought response and its productivity under stress in crop plants. Exploring root architectural traits in mesocosm (seedlings) and field (mature plants) revealed genetic variability for key traits to stabilize productivity in various alfalfa-growing environments. Mesocosm experiments with the difference in the intensity of stress imposed, root volume (Exp. 1), and RW, VRL (Exp. 2) were found to be positively associated with shoot biomass yield. Identifying root traits like median, the maximum number of roots, total root length, max-width, network area, surface area, maximum radius enhancing biomass across multiple harvests is a very promising report in alfalfa. Drought-tolerant maize plants had longer roots with enhanced water capture, stomatal conductance, and plant water status (Gao and Lynch, 2016). Thicker roots may also contribute to their ability to penetrate hard/clay soils to access water in deeper soil layers (Clark et al., 2008) and thus increase adaptation to low-input and rainfed agroecosystems. Certain root traits related to root thickness (like diameter [D], average radius [AR], fine [FRF], medium [MRF] and coarse [CRF] radius frequencies, and FI-fineness index) were negatively associated with NDVI values and biomass yield in alfalfa under rainfed condition. Lateral roots with more branching and smaller diameter (finer roots) were proven to optimize yield under drought stress (Suji et al., 2012; Prince et al., 2016, 2018a,b,^2019^). Recently, the RhizoVision crown platform was used to show that alfalfa compensates for losing the taproot due to cotton root rot by proliferating more fine lateral roots (Mattupalli et al., 2019). The comparative study between greenhouse and field identified root length, volume and root spread in the top soil zone as traits to be associated with alfalfa biomass yield under drought. Specific root traits are critical in the plant’s ability to forage for nutrients and water at deeper soil layers (Lynch, 2013; Prince et al., 2013).

The PI478573 had a highly branched root system with finer roots and smaller leaves, which might be beneficial to capture more water and maintain plant survival under drought. Whereas the PI502521 had a robust root system with enhanced water uptake and lower transpiration loss with bigger leaves, which might prove beneficial in prolonged drought stress productivity. Higher root-to-shoot ratios can increase root water absorption in drought-tolerant alfalfa populations (Avice et al., 1997; Erice et al., 2010). The genotype PI502521 might have positive alleles to improve both leaf root responses, maintain shoot and root ratio to enhance productivity in rainfed conditions. The role of a well-developed and deeper root system in stabilizing yield under water stress was well established in rice (Kato et al., 2007; Suji et al., 2012), chickpea (Kashiwagi et al., 2015), sorghum (Singh et al., 2011), and soybean (Prince et al., 2015b,^2016^, ^2017^). Identification of novel traits from wild accessions or crop landraces successfully improved the productivity of crop species (Brozynska et al., 2017), especially in legumes to derive traits like disease resistance (Dempewolf et al., 2017), drought avoidance (Manavalan et al., 2015; Prince et al., 2015b,^2020^), and tolerance (Prince et al., 2017).

Developing an alfalfa cultivar with optimized yield under drought is possible through poly-cross between diverse genotypes with beneficial leaf and root responses and by integrating multiple drought adaptive traits. The genotypes PI478573 and PI502521 were found to have beneficial alleles for leaf and root traits, respectively, to maintain leaf physiological processes (stomatal conductance and chlorophyll content) and improve productivity under limited water availability. Thus, identifying these useful alleles will enable development of alfalfa cultivars adapted to fluctuating environmental conditions and suitability to wider growing environments.

## Data Availability Statement

The original contributions presented in the study are included in the article/Supplementary Material, further inquiries can be directed to the corresponding author/s.

## Author Contributions

SP compiled the raw data, performed the data and image analysis, interpreted the results, and wrote the manuscript. MA conceived and designed the experiment. CM and TH designed the experiment and collected phenotypic and physiological measurements. FL developed the MATLAB scripts for leaf and image analysis and performed the computational analysis. RM performed the calibration and image processing of leaf images for script-based analysis. PM coordinated the script-based image analysis. AS and LY helped processing of root images with RhizoVision Explorer program. MM and CY edited the manuscript. All the authors read and approved the final manuscript, reviewed the manuscript before submission.

## Conflict of Interest

SP was employed by company BASF. FL was employed by company MLM Medical Labs. MM was employed by company Bayer Crop Science. The remaining authors declare that the research was conducted in the absence of any commercial or financial relationships that could be construed as a potential conflict of interest.

## Publisher’s Note

All claims expressed in this article are solely those of the authors and do not necessarily represent those of their affiliated organizations, or those of the publisher, the editors and the reviewers. Any product that may be evaluated in this article, or claim that may be made by its manufacturer, is not guaranteed or endorsed by the publisher.

## Footnotes

1
http://www.ardmorecity.org

## Abbreviations

CC: chlorophyll content
gs: stomatal conductance
LWP: leaf water potential
OP: osmotic potential
SFW: shoot fresh weight
SDW: shoot dry weight
RFW: root fresh weight
RDW: root dry weight.
