# Supplementary material for: Intraspecific Variation for Leaf Physiological and Root Morphological Adaptation to Drought Stress in Alfalfa (Medicago sativa L.)
Source: Front Plant Sci. 2022 May 3;13:795011. doi: 10.3389/fpls.2022.795011 (PMC9117100; doi:10.3389/fpls.2022.795011)
Supplement: Supplementary file 1 [file Data_Sheet_1.docx]

**Supplementary Table 1.** Characteristics of the two alfalfa populations evaluated in this study and the corresponding phenotypic traits based on data derived from the Germplasm Resource Information Network (GRIN). Corresponding scales for each descriptor are shown in parenthesis.

| **Category** | **Descriptors and scale used** | **PI 478573** | **PI 502521** |
| --- | --- | --- | --- |
|  |  | *Medicago sativa* L. subsp. *sativa* | *Medicago sativa* L. subsp. *varia* |
| Stress | Frost damage  (1 = least, 9 = most) | 8 | 4 |
| Stress | Winter injury  (1 = least, 9 = most) | 9 | 3 |
| Growth | Crown width  (1 = narrow, 5 = wide) | 2 | 3 |
| Growth | Fall growth by plant height (cm) | 16 to 20 | 11 to 15 |
| Morphology | Crown branching  (1 = low, 5 = deep) | 2 | 3 |
| Morphology | Crown bud number  (1 = few, 5 = many) | 1 | 2 |
| Morphology | Depth of the crown  (1 = shallow, 5 = deep) | 3 | 4 |
| Morphology | Fibrous root mass  (1 = none, 5 = many) | 4 | 3 |
| Morphology | Secondary root number  (1 = 0 to 1, 6 = greater than or equal to 12) | 3 | 4 |
| Morphology | Secondary root position  (1 - >= 20 cm below crown, 5 - <= 5 cm below crown) | 1 | 2 |
| Morphology | Unifoliate internode length (mm) | 13.78 | 11.58 |

**Supplementary Table 2**. Experimental conditions for evaluating the three alfalfa populations under well-watered (WW) and water-stressed (WS) treatments.

| **Experiment** | **Date of transplanting (DAT)** | **Date of last irrigation** | **WS Initiation** | **Duration of WS** |
| --- | --- | --- | --- | --- |
| **Exp. 1*** | Sept 1, 2015 | Sept 26, 2015 | From 27 to 55 DAT | 29 days |
| **Exp. 2**** | Sept 1, 2015 | Oct 10, 2015 | From 38 to 69 DAT | 32 days |

* Refers to the mesocosms in Exp. 1: the bottom 61 cm contained all-purpose sand and the top 15 cm contained Metro Mix 360.

** Refers to the mesocosms in Exp. 2: the mesocosm was filled with a sand and perlite mix at a ratio of 2:1 (v/v).

**Supplementary Table 3.** Analysis of variance of leaf morphological traits in the three alfalfa grown in the greenhouse under water stress (WS) in experimental conditions of Exp. 1 and 2. Means with the different letter across cultivars indicates significant difference between them based on LSD (α=0.05) value.

| **Leaf Trait** | **Leaflet position** | **PI478573** | **PI502521** | **Bulldog805** | **LSD (α=0.05)** | **Genotype** |
| --- | --- | --- | --- | --- | --- | --- |
| **Experiment 1** | | | | | | |
| Total Blade Area | Middle | 187,694b | 279,247a | 264,485a | 64,692 | * |
|  | Left | 153,309b | 223,850a | 214,214a | 54,731 | * |
| Blade area/length | Left | 0.99b | 1.23a | 1.19a | 0.19 | * |
| Blade width | Middle | 308.87b | 434.44a | 418.55a | 67.20 | * |
|  | Left | 279.49b | 384.61a | 361.51a | 67.71 | * |
|  | Right | 281.10b | 387.15a | 363.95a | 80.04 | * |
| Length to width Ratio | Middle | 2.76a | 2.08b | 2.15b | 0.41 | * |
|  | Left | 2.77a | 2.11b | 2.29b | 0.39 | * |
|  | Right | 2.87a | 2.05b | 2.32b | 0.46 | * |
| **Experiment 2** | | | | | | |
| Total Blade Area | Middle | 155,290b | 190,214ab | 230,568a | 62,960 | * |
| Blade width | Middle | 292.74b | 372.14a | 420.53a | 70.13 | ** |
|  | Left | 247.49b | 333.98a | 337.18a | 67.45 | * |
|  | Right | 254.75b | 355.20a | 334.66a | 64.05 | ** |
| Entropy | Left | 1.51a | 1.13b | 1.30ab | 0.26 | * |
| Compactness | Middle | 0.38b | 0.45a | 0.45a | 0.05 | ** |
|  | Left | 0.35b | 0.45a | 0.42a | 0.06 | * |
|  | Right | 0.34b | 0.45a | 0.34b | 0.07 | ** |
| Length to width ratio | Middle | 2.61a | 1.92b | 1.82b | 0.26 | *** |
|  | Left | 2.89a | 2.08b | 2.05b | 0.42 | *** |
|  | Right | 2.84a | 1.98b | 2.21b | 0.52 | ** |
