# Supplementary material for: Intraspecific Variation for Leaf Physiological and Root Morphological Adaptation to Drought Stress in Alfalfa (Medicago sativa L.)
Source: Front Plant Sci. 2022 May 3;13:795011. doi: 10.3389/fpls.2022.795011 (PMC9117100; doi:10.3389/fpls.2022.795011)
Supplement: Supplementary file 2 [file Presentation_1.PPTX]

## Slide 1
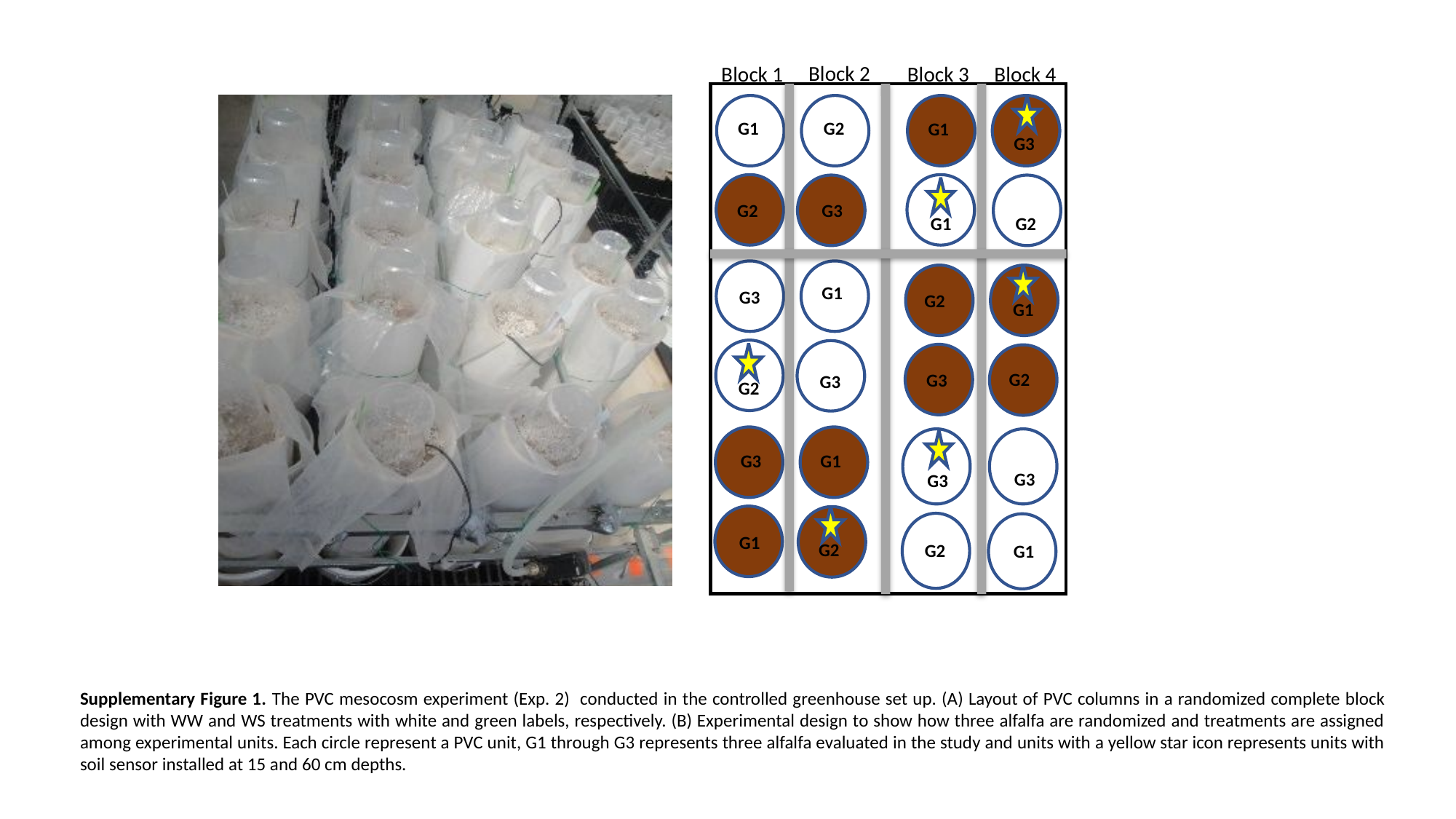

Block 2
Block 1
Block 3
Block 4
G2
G1
G1
G3
G2
G3
G2
G1
G1
G3
G2
G1
G2
G3
G3
G2
G3
G1
G3
G3
G1
G2
G2
G1
Supplementary Figure 1. The PVC mesocosm experiment (Exp. 2) conducted in the controlled greenhouse set up. (A) Layout of PVC columns in a randomized complete block design with WW and WS treatments with white and green labels, respectively. (B) Experimental design to show how three alfalfa are randomized and treatments are assigned among experimental units. Each circle represent a PVC unit, G1 through G3 represents three alfalfa evaluated in the study and units with a yellow star icon represents units with soil sensor installed at 15 and 60 cm depths.

## Slide 2
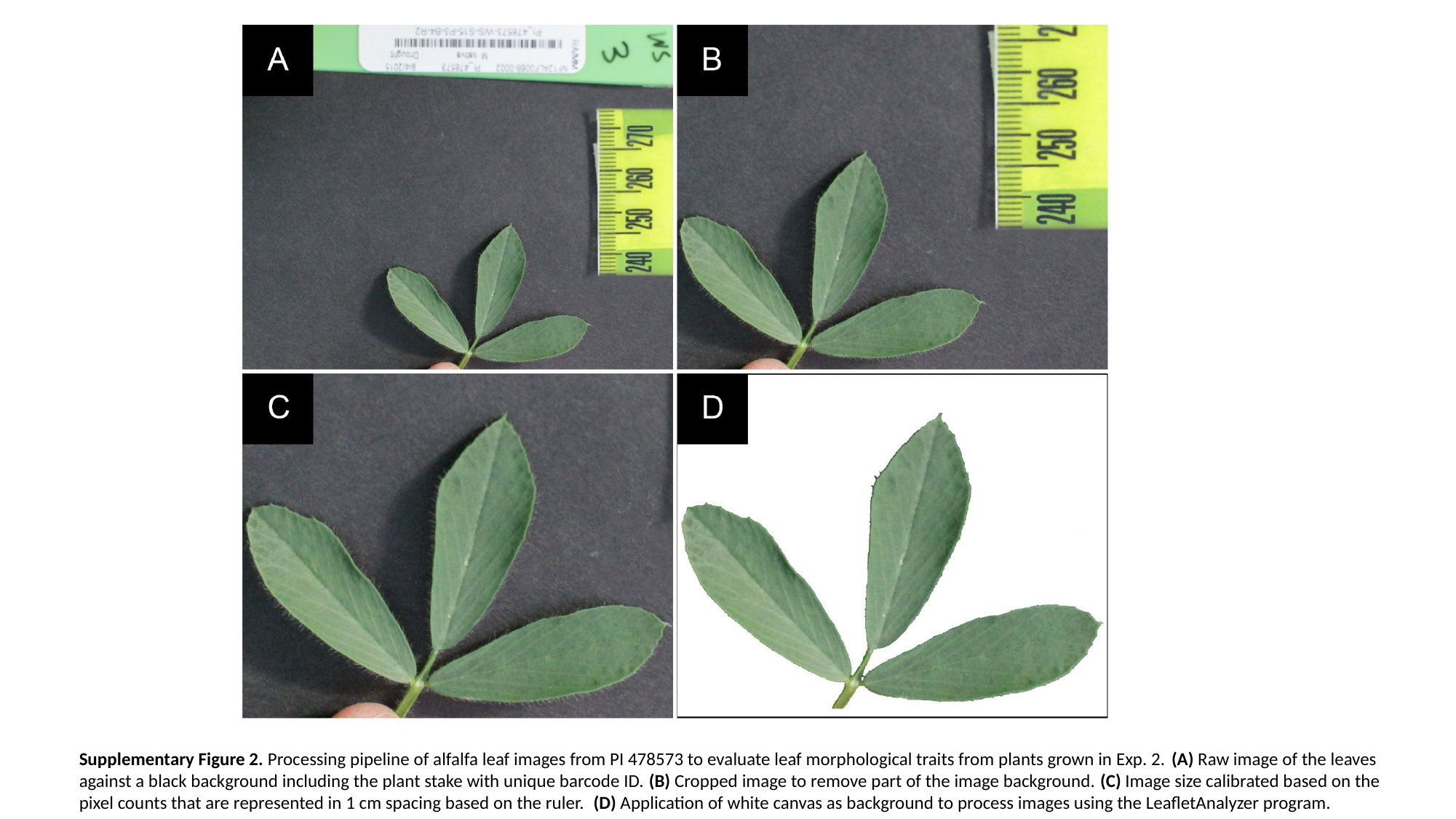

Supplementary Figure 2. Processing pipeline of alfalfa leaf images from PI 478573 to evaluate leaf morphological traits from plants grown in Exp. 2. (A) Raw image of the leaves against a black background including the plant stake with unique barcode ID. (B) Cropped image to remove part of the image background. (C) Image size calibrated based on the pixel counts that are represented in 1 cm spacing based on the ruler. (D) Application of white canvas as background to process images using the LeafletAnalyzer program.

## Slide 3
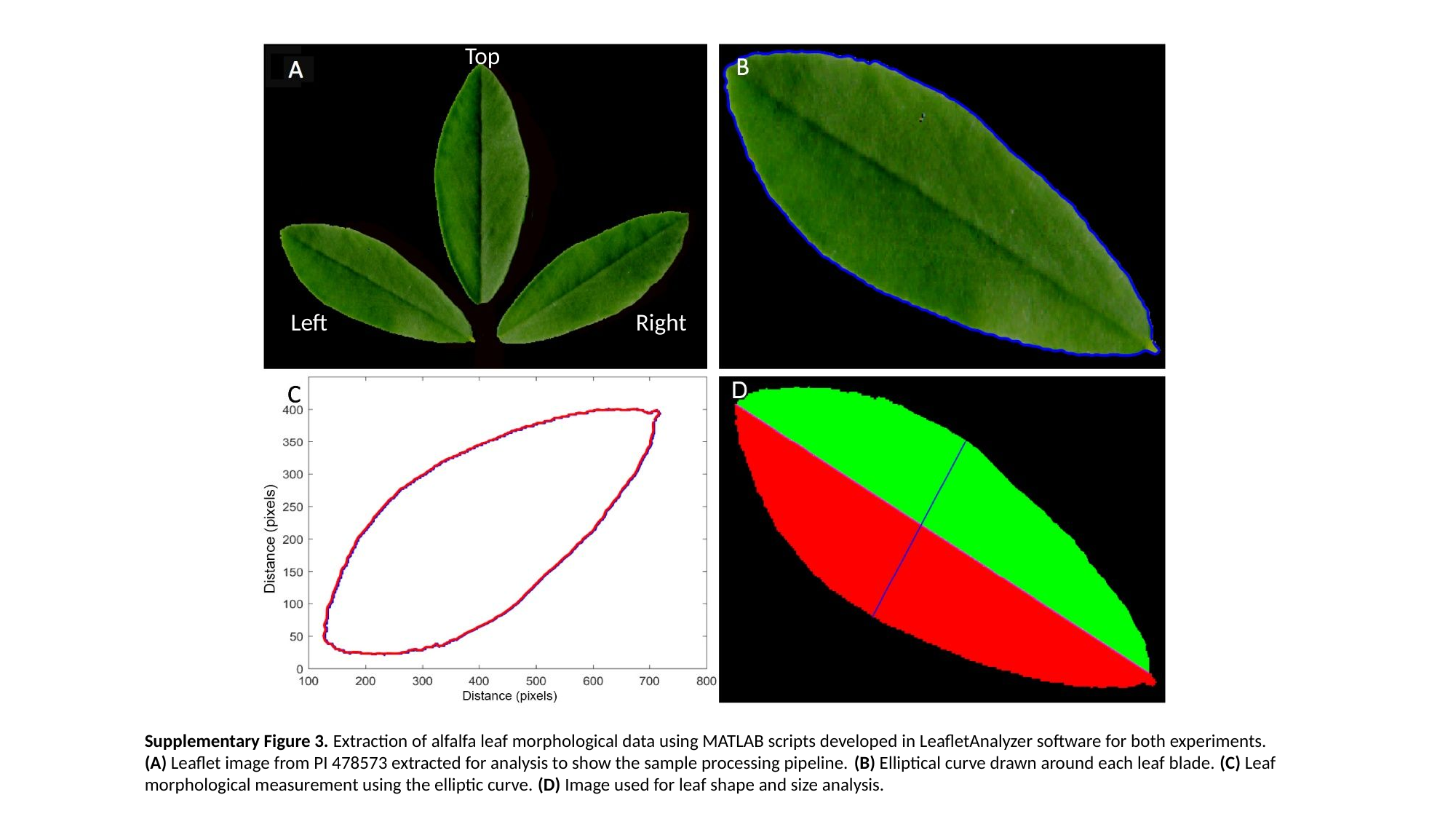

B
A
Top
Left
Right
D
Supplementary Figure 3. Extraction of alfalfa leaf morphological data using MATLAB scripts developed in LeafletAnalyzer software for both experiments. (A) Leaflet image from PI 478573 extracted for analysis to show the sample processing pipeline. (B) Elliptical curve drawn around each leaf blade. (C) Leaf morphological measurement using the elliptic curve. (D) Image used for leaf shape and size analysis.

## Slide 4
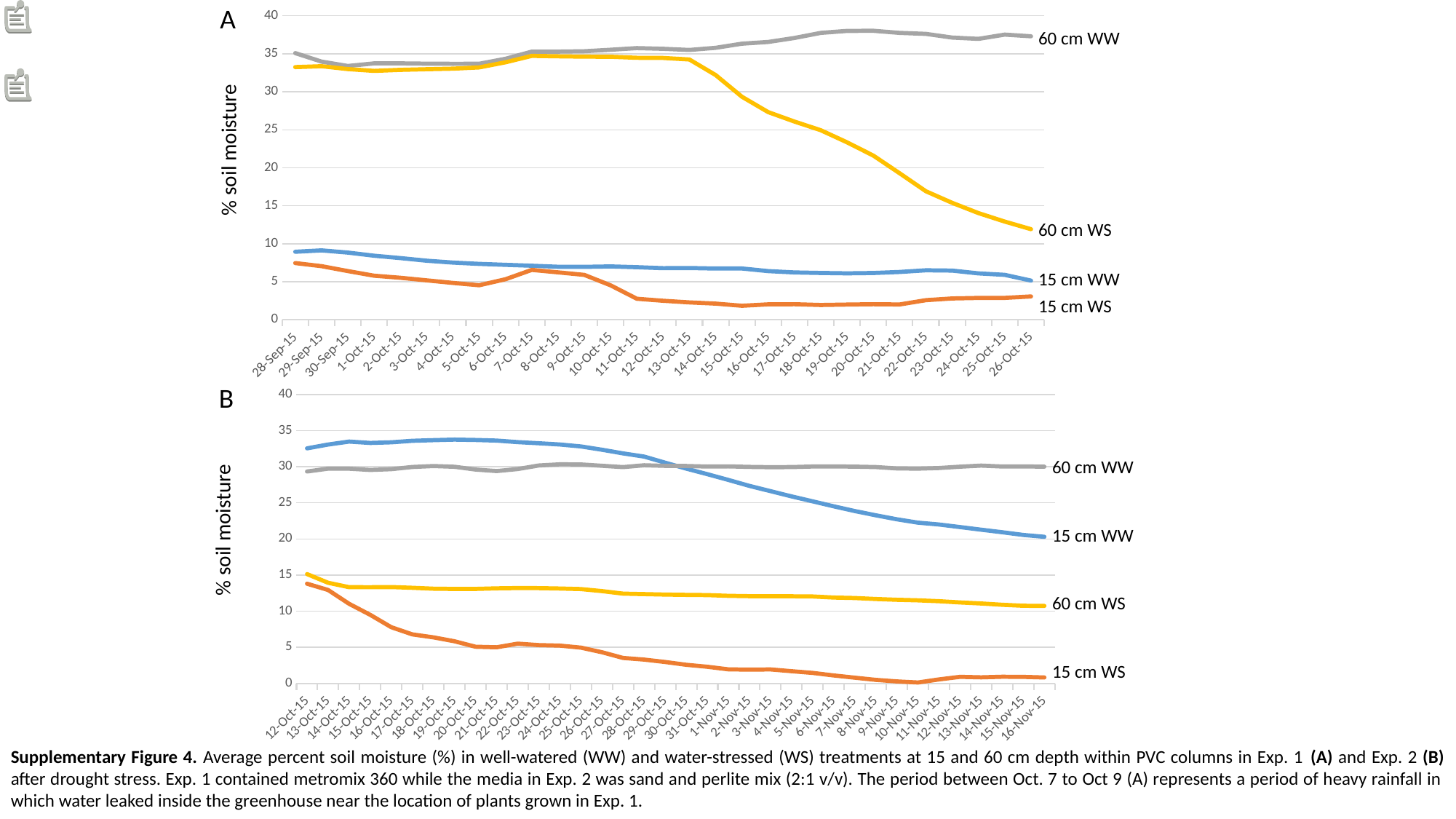

A
### Chart
| Category | WW-15cm | WS-15cm | WW-60cm | WS-60cm |
|---|---|---|---|---|
| 28-Sep-15 | 8.95209895679727 | 7.46092291859289 | 35.0905731320381 | 33.2515377085656 |
| 29-Sep-15 | 9.13083649162824 | 7.05480413356175 | 33.9744062783817 | 33.3676790352911 |
| 30-Sep-15 | 8.84293326719975 | 6.41300833861654 | 33.3978461101651 | 32.9868252544353 |
| 1-Oct-15 | 8.43323230122526 | 5.80251042265445 | 33.743631405135 | 32.753222156316 |
| 2-Oct-15 | 8.11685940328365 | 5.53063336216534 | 33.7440085286895 | 32.8876521593581 |
| 3-Oct-15 | 7.77484473461906 | 5.18673333572224 | 33.7006436971327 | 32.9698565881699 |
| 4-Oct-15 | 7.52747813239694 | 4.85188335878775 | 33.6821663814286 | 33.0437647023549 |
| 5-Oct-15 | 7.35797918556879 | 4.54342917461569 | 33.6908398196101 | 33.2110012260576 |
| 6-Oct-15 | 7.22863952590463 | 5.33530427298198 | 34.3488502626618 | 33.8818326437225 |
| 7-Oct-15 | 7.11212089130034 | 6.56572710722685 | 35.2881651371717 | 34.7242368074755 |
| 8-Oct-15 | 6.97467375624304 | 6.23917294821391 | 35.2843940568467 | 34.6699365259459 |
| 9-Oct-15 | 6.96618942699085 | 5.90733953674013 | 35.3356780173878 | 34.6216697711498 |
| 10-Oct-15 | 7.02199792334189 | 4.53324788832106 | 35.5362852104008 | 34.6135625305275 |
| 11-Oct-15 | 6.92188215907663 | 2.76849794706019 | 35.7466982677579 | 34.4740416388959 |
| 12-Oct-15 | 6.79159997574364 | 2.50416250394968 | 35.6614778128763 | 34.4536788295954 |
| 13-Oct-15 | 6.81384784790377 | 2.28696247407546 | 35.5064955229561 | 34.2530706586937 |
| 14-Oct-15 | 6.74521867573882 | 2.12670209778783 | 35.7896860378484 | 32.205319730565 |
| 15-Oct-15 | 6.75162920185054 | 1.84577500913292 | 36.3341935910285 | 29.3457081308588 |
| 16-Oct-15 | 6.40414689357082 | 2.03243125773345 | 36.5600666962564 | 27.3273694949845 |
| 17-Oct-15 | 6.23031147988513 | 2.05203956963184 | 37.0823270330826 | 26.0809208809709 |
| 18-Oct-15 | 6.16073963271144 | 1.94645624530191 | 37.7516498168309 | 24.9474085013693 |
| 19-Oct-15 | 6.11398118004824 | 2.00226458837278 | 38.0137233374019 | 23.3495176459352 |
| 20-Oct-15 | 6.15809995991488 | 2.04751458950341 | 38.0288069136441 | 21.6079583236327 |
| 21-Oct-15 | 6.29177597002126 | 2.01470835211997 | 37.7490104486545 | 19.2860677372664 |
| 22-Oct-15 | 6.51406664207267 | 2.58033333035807 | 37.624195497483 | 16.9168530536505 |
| 23-Oct-15 | 6.47390732968537 | 2.81073123915121 | 37.1339874962966 | 15.3943792645199 |
| 24-Oct-15 | 6.10606249926301 | 2.87370414783557 | 36.9639228408535 | 14.0368791646324 |
| 25-Oct-15 | 5.91111041527862 | 2.88162291205178 | 37.5265308655798 | 12.9195812740363 |
| 26-Oct-15 | 5.1527959042384 | 3.07582080325422 | 37.3074455186725 | 11.9227613322437 |60 cm WW
% soil moisture
60 cm WS
15 cm WW
15 cm WS
B
### Chart
| Category | WW15cm | WS-15cm | WW-60cm | WS-60cm |
|---|---|---|---|---|
| 12-Oct-15 | 32.5343248744806 | 13.800447996861 | 29.3326990911737 | 15.1341914199293 |
| 13-Oct-15 | 33.0611107560496 | 12.9365501265662 | 29.7244881046936 | 13.9335582343241 |
| 14-Oct-15 | 33.4732627185682 | 11.018327010485 | 29.7182662334914 | 13.3192896532516 |
| 15-Oct-15 | 33.2832122221589 | 9.49528752050052 | 29.5470709912479 | 13.3143874661376 |
| 16-Oct-15 | 33.3699413885673 | 7.78219789693443 | 29.6518999384716 | 13.3404061819116 |
| 17-Oct-15 | 33.5795993295809 | 6.79178859475845 | 29.9596003101518 | 13.2401022439202 |
| 18-Oct-15 | 33.6689682056506 | 6.37624272203539 | 30.0904482292632 | 13.1141564808786 |
| 19-Oct-15 | 33.7462699661652 | 5.83607080382838 | 29.9844872982552 | 13.0772021599114 |
| 20-Oct-15 | 33.6912161360184 | 5.08548644914602 | 29.5974118712669 | 13.0843666382134 |
| 21-Oct-15 | 33.6090121418238 | 5.00347082464335 | 29.3905815264831 | 13.161668771257 |
| 22-Oct-15 | 33.3974686451256 | 5.51083649916109 | 29.6733937303846 | 13.1997542611013 |
| 23-Oct-15 | 33.2406017308434 | 5.30589171855051 | 30.1636021429052 | 13.1903271035602 |
| 24-Oct-15 | 33.0652579354743 | 5.23990211901643 | 30.3227309137583 | 13.1371583789587 |
| 25-Oct-15 | 32.7997918861608 | 4.95614687994627 | 30.3106646208713 | 13.0575940789034 |
| 26-Oct-15 | 32.3408812905351 | 4.32057285597693 | 30.1234426054483 | 12.7770439799254 |
| 27-Oct-15 | 31.8367208043734 | 3.53039474523484 | 29.9369751475751 | 12.4259791880225 |
| 28-Oct-15 | 31.3951558743914 | 3.30339061352637 | 30.1941460929811 | 12.3622519817824 |
| 29-Oct-15 | 30.5497355759144 | 2.96967188881657 | 30.1100565353408 | 12.2955083381385 |
| 30-Oct-15 | 29.7295788613458 | 2.58221875701565 | 30.0953500904143 | 12.2540293261409 |
| 31-Oct-15 | 28.9625918182234 | 2.31015313438547 | 30.0189906964079 | 12.224993792673 |
| 1-Nov-15 | 28.164306593438 | 1.95399792925552 | 30.041050290068 | 12.1243125371014 |
| 2-Nov-15 | 27.3400020164748 | 1.91402708078385 | 29.970347112976 | 12.0764230921244 |
| 3-Nov-15 | 26.613362506032 | 1.94268543491489 | 29.926416852201 | 12.0752918766812 |
| 4-Nov-15 | 25.8870999018351 | 1.69588437456696 | 29.9428200271601 | 12.0583231638496 |
| 5-Nov-15 | 25.2023166355987 | 1.45964165058103 | 30.0201219817003 | 12.0345668246349 |
| 6-Nov-15 | 24.5160249372323 | 1.10706874596265 | 30.0344511711349 | 11.8882582522929 |
| 7-Nov-15 | 23.8598999877771 | 0.799745840049581 | 30.0065470005696 | 11.8218915925051 |
| 8-Nov-15 | 23.2686334600051 | 0.498079158811985 | 29.9464018937821 | 11.6838791640475 |
| 9-Nov-15 | 22.7177146511773 | 0.28314166944862 | 29.7523927409202 | 11.5779187607889 |
| 10-Nov-15 | 22.2569187792639 | 0.136267717607552 | 29.7269393922761 | 11.4979770267382 |
| 11-Nov-15 | 21.9963544203589 | 0.56029792613117 | 29.8125372190649 | 11.3791957109546 |
| 12-Nov-15 | 21.6415187188735 | 0.920035424011682 | 29.9999479437247 | 11.2091312572981 |
| 13-Nov-15 | 21.2780103087425 | 0.845372932963073 | 30.1455024940272 | 11.0650854573275 |
| 14-Nov-15 | 20.9288311501344 | 0.93247917733 | 30.0229501444846 | 10.8874791534618 |
| 15-Nov-15 | 20.5555188314368 | 0.913059390101504 | 30.0340740863855 | 10.7498438407977 |
| 16-Nov-15 | 20.2977189794183 | 0.832175012005286 | 29.9998222746783 | 10.7358916041752 |60 cm WW
% soil moisture
15 cm WW
60 cm WS
15 cm WS
Supplementary Figure 4. Average percent soil moisture (%) in well-watered (WW) and water-stressed (WS) treatments at 15 and 60 cm depth within PVC columns in Exp. 1 (A) and Exp. 2 (B) after drought stress. Exp. 1 contained metromix 360 while the media in Exp. 2 was sand and perlite mix (2:1 v/v). The period between Oct. 7 to Oct 9 (A) represents a period of heavy rainfall in which water leaked inside the greenhouse near the location of plants grown in Exp. 1.

## Slide 5
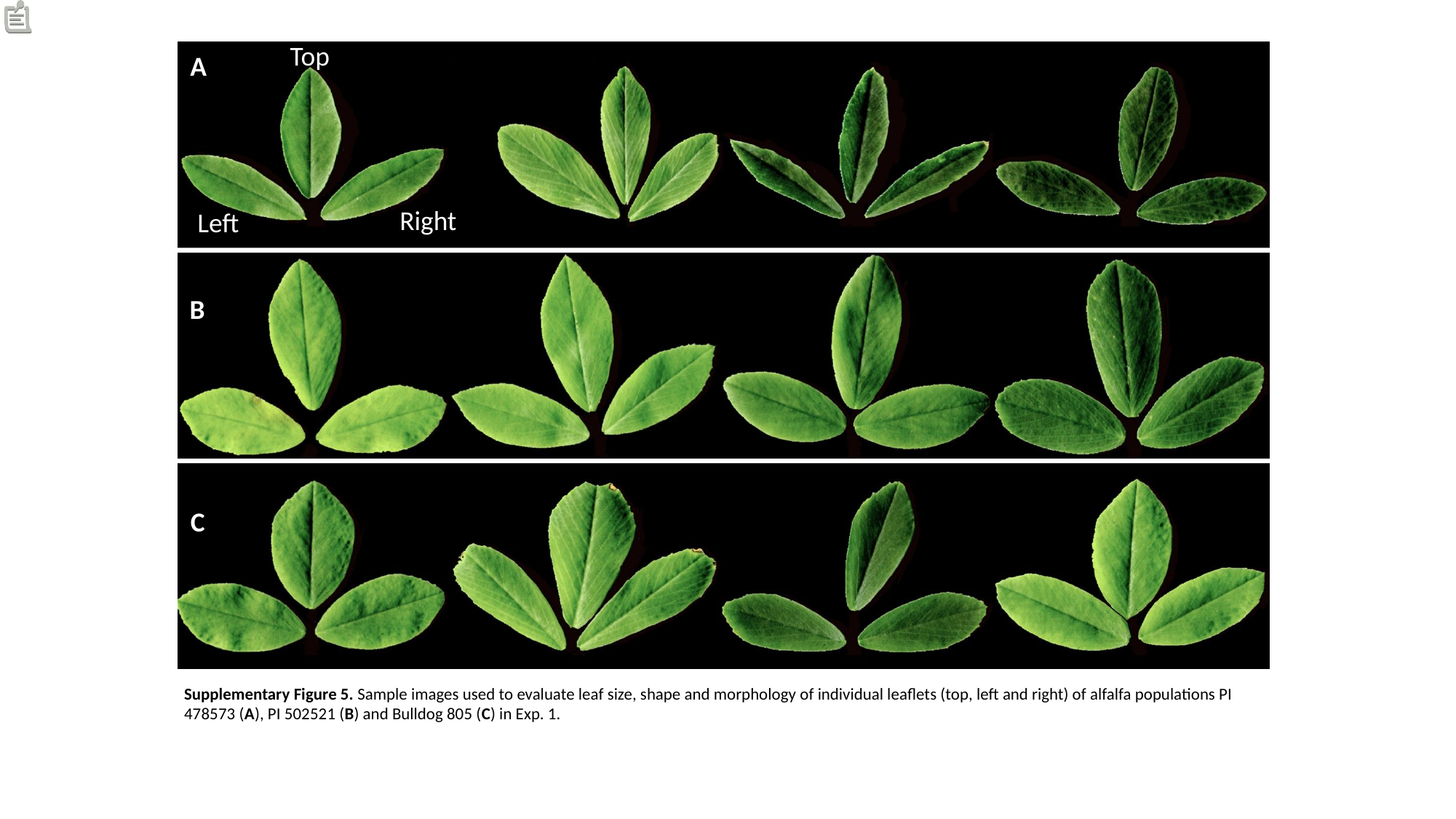

Top
A
Right
Left
B
C
Supplementary Figure 5. Sample images used to evaluate leaf size, shape and morphology of individual leaflets (top, left and right) of alfalfa populations PI 478573 (A), PI 502521 (B) and Bulldog 805 (C) in Exp. 1.

## Slide 6
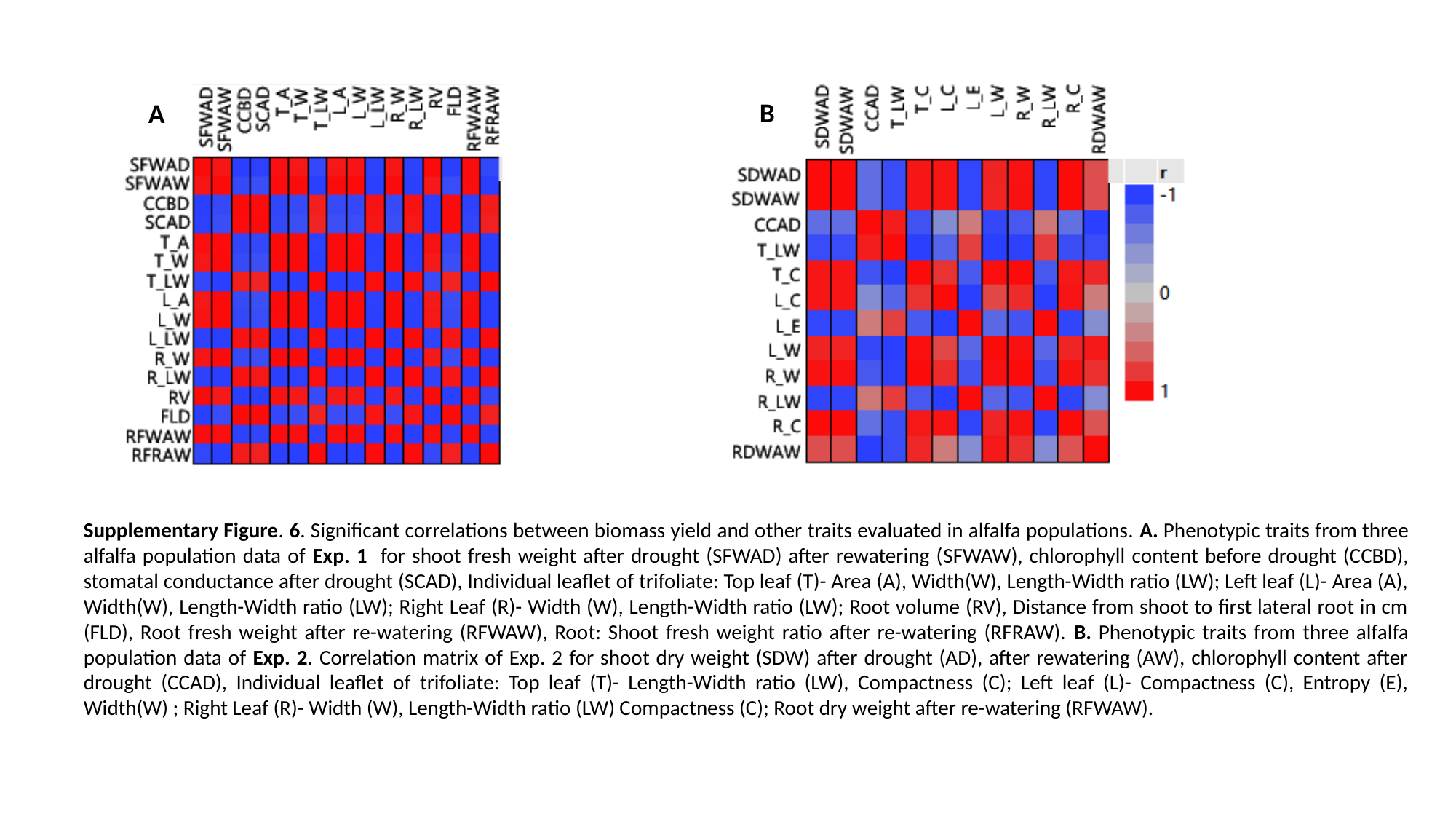

B
A
Supplementary Figure. 6. Significant correlations between biomass yield and other traits evaluated in alfalfa populations. A. Phenotypic traits from three alfalfa population data of Exp. 1 for shoot fresh weight after drought (SFWAD) after rewatering (SFWAW), chlorophyll content before drought (CCBD), stomatal conductance after drought (SCAD), Individual leaflet of trifoliate: Top leaf (T)- Area (A), Width(W), Length-Width ratio (LW); Left leaf (L)- Area (A), Width(W), Length-Width ratio (LW); Right Leaf (R)- Width (W), Length-Width ratio (LW); Root volume (RV), Distance from shoot to first lateral root in cm (FLD), Root fresh weight after re-watering (RFWAW), Root: Shoot fresh weight ratio after re-watering (RFRAW). B. Phenotypic traits from three alfalfa population data of Exp. 2. Correlation matrix of Exp. 2 for shoot dry weight (SDW) after drought (AD), after rewatering (AW), chlorophyll content after drought (CCAD), Individual leaflet of trifoliate: Top leaf (T)- Length-Width ratio (LW), Compactness (C); Left leaf (L)- Compactness (C), Entropy (E), Width(W) ; Right Leaf (R)- Width (W), Length-Width ratio (LW) Compactness (C); Root dry weight after re-watering (RFWAW).

## Slide 7
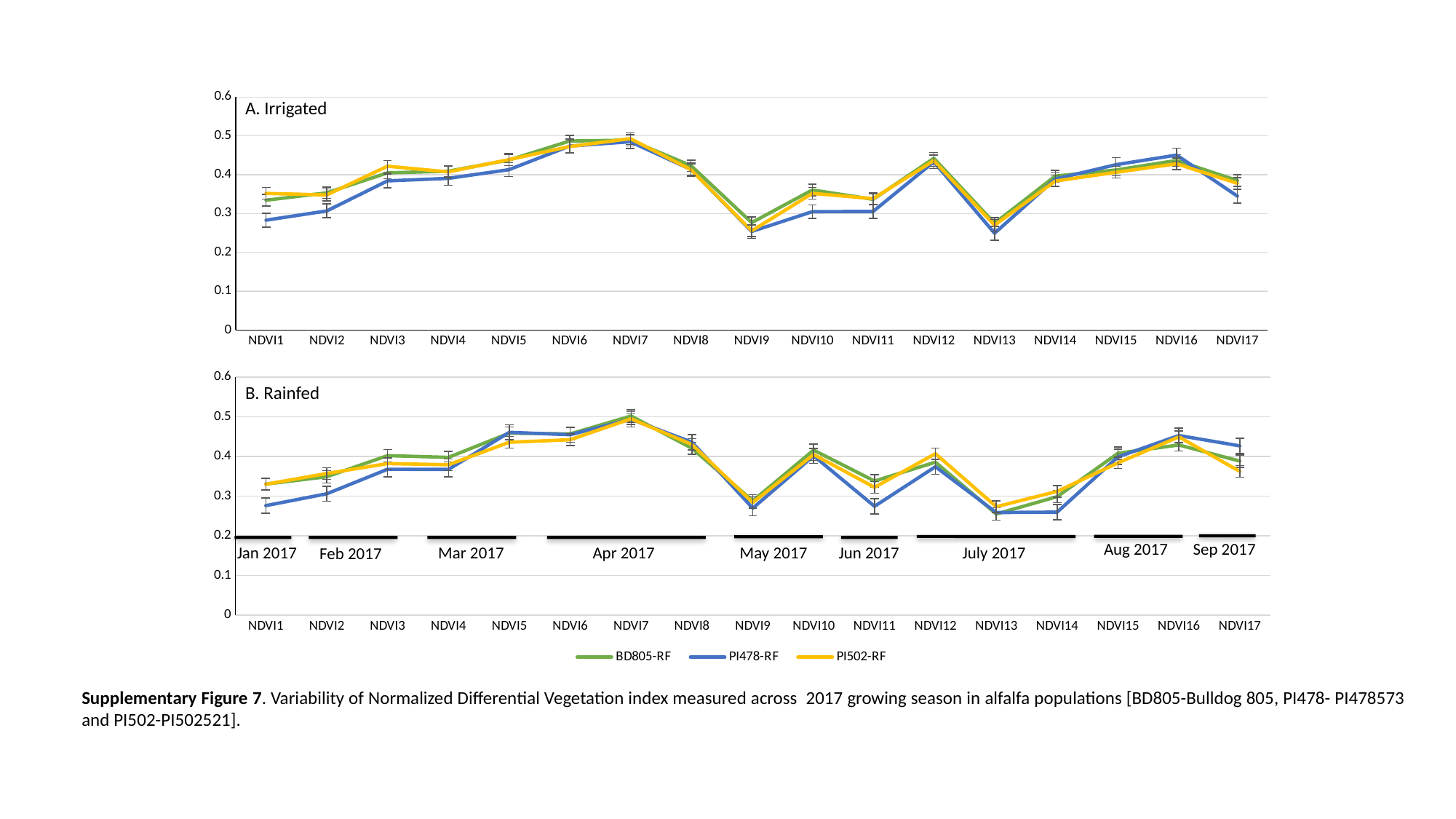

### Chart
| Category | BD805-IR | PI478-IR | PI502-IR |
|---|---|---|---|
| NDVI1 | 0.334367536963999 | 0.282993247024691 | 0.351894601137897 |
| NDVI2 | 0.3534177070766 | 0.306848820240974 | 0.348048426882211 |
| NDVI3 | 0.404787996202272 | 0.384202569229654 | 0.421949700047002 |
| NDVI4 | 0.409040695146013 | 0.390589621212677 | 0.407286165370662 |
| NDVI5 | 0.437795717503153 | 0.412963622420535 | 0.438942466706824 |
| NDVI6 | 0.486872618052677 | 0.473808765091068 | 0.472581726182376 |
| NDVI7 | 0.489127176757752 | 0.485016363543278 | 0.493102480636621 |
| NDVI8 | 0.423290686405212 | 0.413494920166947 | 0.412944207126227 |
| NDVI9 | 0.276738075625674 | 0.253790286211201 | 0.255400635064641 |
| NDVI10 | 0.360816526069432 | 0.304949240228795 | 0.352025146753014 |
| NDVI11 | 0.336631390377998 | 0.30551041814082 | 0.338445834993732 |
| NDVI12 | 0.442309418011634 | 0.433272293731739 | 0.436363708549097 |
| NDVI13 | 0.274818465567437 | 0.249188833752659 | 0.269994259732931 |
| NDVI14 | 0.396456342155937 | 0.388987448888315 | 0.38378798425363 |
| NDVI15 | 0.412203489284994 | 0.425910882096377 | 0.406275550747687 |
| NDVI16 | 0.436790624551725 | 0.45062377697599 | 0.428160906705703 |
| NDVI17 | 0.384987790842057 | 0.344899525098411 | 0.377934255096292 |
### Chart
| Category | BD805-RF | PI478-RF | PI502-RF |
|---|---|---|---|
| NDVI1 | 0.33052755418171 | 0.27629070696275 | 0.330197478357994 |
| NDVI2 | 0.349040188307074 | 0.306017958713331 | 0.357221238107585 |
| NDVI3 | 0.402267251004871 | 0.367854852156565 | 0.382176622608446 |
| NDVI4 | 0.397846793145535 | 0.367449267191088 | 0.37928910923996 |
| NDVI5 | 0.458888188176277 | 0.460885379647254 | 0.435866692467942 |
| NDVI6 | 0.456896221047474 | 0.454913733715209 | 0.442251900124929 |
| NDVI7 | 0.502108406405105 | 0.493726994743042 | 0.49497668164893 |
| NDVI8 | 0.420728033288652 | 0.436025334436523 | 0.430298626641582 |
| NDVI9 | 0.288261293640976 | 0.269937022173347 | 0.284124018323949 |
| NDVI10 | 0.41604704725191 | 0.401802627507103 | 0.404929933371308 |
| NDVI11 | 0.338247978537337 | 0.274565914761891 | 0.322406059109958 |
| NDVI12 | 0.385801501396398 | 0.374335502976353 | 0.407122617306906 |
| NDVI13 | 0.255219914311558 | 0.258873492488203 | 0.273630850000514 |
| NDVI14 | 0.298865219462366 | 0.259902414866803 | 0.312131012880502 |
| NDVI15 | 0.408290257480309 | 0.399189176166184 | 0.384090373067631 |
| NDVI16 | 0.429115366876775 | 0.452850004114442 | 0.449424958748732 |
| NDVI17 | 0.388499512557438 | 0.426831479446109 | 0.362461674695236 |Sep 2017
Aug 2017
July 2017
Mar 2017
Apr 2017
May 2017
Jun 2017
Feb 2017
Jan 2017
A. Irrigated
B. Rainfed
Supplementary Figure 7. Variability of Normalized Differential Vegetation index measured across 2017 growing season in alfalfa populations [BD805-Bulldog 805, PI478- PI478573 and PI502-PI502521].

## Slide 8
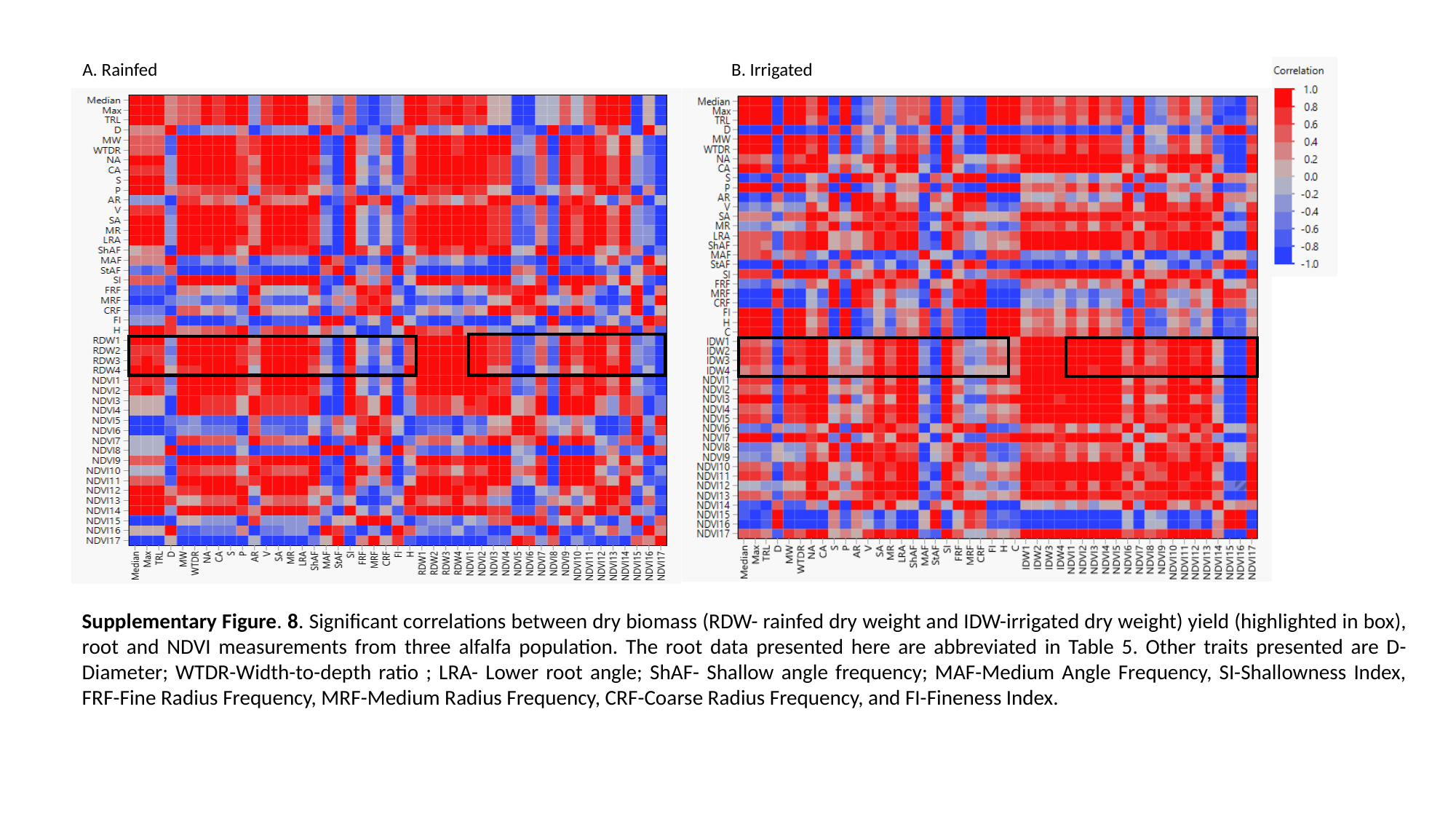

A. Rainfed
B. Irrigated
Supplementary Figure. 8. Significant correlations between dry biomass (RDW- rainfed dry weight and IDW-irrigated dry weight) yield (highlighted in box), root and NDVI measurements from three alfalfa population. The root data presented here are abbreviated in Table 5. Other traits presented are D- Diameter; WTDR-Width-to-depth ratio ; LRA- Lower root angle; ShAF- Shallow angle frequency; MAF-Medium Angle Frequency, SI-Shallowness Index, FRF-Fine Radius Frequency, MRF-Medium Radius Frequency, CRF-Coarse Radius Frequency, and FI-Fineness Index.
